# Epithelial Carcinoma of the Left Superior Maxilla, with Secondary Deposits in the Lungs

**Published:** 1882-04

**Authors:** Frank V. Walton


### CASE DEPARTMENT.

### I.—EPITHELIAL CARCINOMA OF THE LEFT SUPERIOR MAXILLA, WITH SECONDARY DEPOSITS IN THE LUNGS.

REPORTED BY FRANK V. WALTON, D.V.S.

The horse from which this neoplasm was removed was a large gray gelding, aet 12.

He first came under personal observation suffering from very severe "quarter crack," which was then discharging pus quite freely. For this he was treated, and a reasonably speedy recovery followed.

From the time of recovery from above disease until the 1st of January, 1882, he remained apparently in the best of health. About this date, a slight enlargement was first noticed, situated at about the centre of the spine of the superior maxillary bone. There was also a slight, but quite offensive discharge from the mouth and left nostril. January 10th, the animal was first seen and examined. I found that there was quite a large tumor involving the left side of the superior jawbone. It was most prominent about midway between the orbit and the infra orbital oramen. The base of the enlargement encroached upon and probably involved to a slight extent the molar and nasal bones of the same side. The principal bone implicated, however, was the maxillary. The center of the growth projected outward beyond the surface about five centimetres and was some nine centimetres in circumference at the base. The whole mass was of stony hardness, and appeared somewhat like an osteoma. Except for the discharge from the mouth and nostril, at this time, it was also observed that the third, fourth and fifth molar teeth of the same side were loose in their sockets. The posterior and middle third of the hard palate had a suspicious look, as if it was becoming invaded by a growth or bone disease, originating superior to it—possibly in the antrum, which might also be the cause of the external protrusion.

A few days later, another examination was made, and it was found that the tumor had gained rapidly in size, and that the condition of the palate was still more unfavorable. The horse had rapidly lost flesh and strength, was unable to eat any solid food, and with great difficulty even semi-solid. He also gave every evidence of suffering great pain.

The question of diagnosis and the advisability of an operation were carefully considered. The principal question of importance in relation to diagnosis was as to its being malignant in nature or not. There was a possibility of its being a simple osteoma, an abscess, a cystic tumor, or some one of the various forms of non-malignant disease of the antrum. If simple in nature, operative interference might be of great service in relieving the sufferings of the horse, and making him eventually useful for several years. From the general history, the rapidity of growth and the suspicious looking mouth, it appeared more like a sarcoma or a carcinoma, the former on account of the rapidity of the growth, and the latter from its extreme hardness.

With all these unfavorable possibilities clearly in view, it was considered advisable to operate, hoping that it might be non-malignant, and the final result good. If malignant, it was certain that the animal would be greatly relieved; and, possibly, by getting well behind the whole diseased mass, arrest its progress, thus temporarily curing the case and enabling him to work for several months or more before it would return.

The owners being informed of all the dangers attending the operation, accompanied with an unfavorable prognosis, gave their consent.

The animal was cast on January 17, 1882, and placed under the influence of chloroform and ether. A crucial incision was made directly over the most prominent part of the growth, and the four angles of integument dissected back. Just underneath and covering in the tumor, was a dense layer of fibrous tissue, giving the tumor the appearance of being encapsulated. An attempt was made to dissect around the capsule, but this brought the operators directly down upon the bone. Cutting through the capsule revealed the fact that the tumor sprang from within the antrum; that the bone forming the external wall of the superior maxillary sinus had become softened, and at points was entirely wanting. A large portion of the bone was very soft and easily cut with a bone chisel, under pressure of the hand. It now became evident that the greater portion of the superior maxillary bone was implicated, and an attempt was made to remove the whole of the diseased portion. On extending the crucial incisions, several enlarged facial branches of the inferior maxillary artery were divided, which gave rise to considerable hemorrhage, and took some time to secure.

The hard and fibrous capsules was cut through, when a softer form of tissue was met with, protruding from the cavity of the antrum. All of the growth protruding beyond the surface of the bone was removed, and then an attempt was made to remove the contents of the superior maxillary sinus and the diseased bone.

The growth being much greater in extent than was first supposed, it became apparent that nothing short of excision of one maxillary bone would carry us well back of the disease.

This being a very serious matter, the operation having already occupied one hour, it was thought best to remove one of the loosened teeth with the alveolar wall, and in that way establish good drainage. The third moral was easily removed from the superior opening. The first and second were quite loose, and were left to be removed in a few days. About one hour after the operation the horse rose to his feet, but suffered quite severely from shock. The following day, however, he was feeling very much better, and was evidently suffering very much less than he had previously. The growth was examined at the Laboratory by Dr. Porter and Mr. Le Boutillier, who found that the neoplasm presented the characteristic features of an epithelial carcinoma, or epithelial masses, in the form of bird-nests or epithelial perles.

During the first week after the operation the horse seemed to improve, suffered less pain, and could eat fairly well. The discharge from the mouth, however, was quite bad, but that from the nostrils entirely ceased. From the open cavity of the antrum a fungus ulcer seemed to be developing, and the palatel processes became more and more implicated.

At the end of a week or ten days numerous enlargements were noticed over the surface of the body, which would easily lead one to suppose that they were farcy buds. These apparent farcy buds rapidly disappeared, and there was no positive evidence of glanders, and the fact of the original growth being carcinomatous in character would exclude the probability of glanders.

From this time until the day of death, although the horse was suffering comparatively little, he evidently was slowly and gradually failing, and a permanent recovery was utterly impossible, and on February 13th, it was decided that it was best to kill the animal.

The animal accordingly was killed, and a necropsy made by Dr. John Wallace. Most of the visceral organs presented the normal appearances, but the lungs were deeply congested and œdematous. They were also thickly studded with small nodules. These masses were carefully examined by Mr. Wm. G. Le Boutillier, who found that they also gave positive evidence of epithelial carcinoma in the shape of epithelial nests, the centre of which failed to take the staining fluid, and the corpuscular elements surrounding the same were arranged in concentric layers, that took the staining matter quite deeply.

The head of the horse was removed, and a careful examination showed that nearly the whole of the middle and posterior third of the palatel processes had been destroyed, and that the growth had also nearly occluded the left nasal cavity, and there was marked evidence of its passing through the septum. This condition would readily show that all operative interference was unable to completely relieve the animal from the cancerous growth.

New York City, March, 1882.

[To the veterinary surgeon this case presents many points worthy of careful consideration.

The result of this operation proves positively that the sufferings of the lower animals can be greatly relieved by scientific surgical interference. Were it possible to determine without doubt, before operating, that the disease was malignant and extensive, on human and other grounds, we would advise the killing of the animal. Had it been non-malignant, a good recovery would probably have been the result. Another fact to be observed is that the surgeon cannot always determine the extent to which the growth may have spread.

The special point of pathological interest is the marked epithelial type of the metastatic deposits in the lungs. In connection with a similar disease, located in the same region in the noted horse Prospero, there are leading points of difference.—Ed.]

